# Detection and Degradation Characterization of 16 Quinolones in Soybean Sprouts by Ultra-High Performance Liquid Chromatography-Tandem Mass Spectrometry

**DOI:** 10.3390/foods11162500

**Published:** 2022-08-18

**Authors:** Hao Deng, Yujie Feng, Guang Wu, Ronghu Zhang, Bei Li, Qingchun Yin, Lin Luo

**Affiliations:** 1 Key Laboratory of Tropical Fruit and Vegetable Cold-Chain of Hainan Province/Institute of Agro-Products of Processing and Design, Hainan Academy of Agricultural Sciences, Haikou 571100, China; 2 Sanya Institute of Hainan Academy of Agricultural Sciences, Sanya 572025, China; 3 Hainan Key Laboratory for Control of Plant Diseases and Pests/Institute of Plant Protection, Hainan Academy of Agricultural Sciences (Research Center of Quality Safety and Standards for Agricultural Products of Hainan Academy of Agricultural Science), Haikou 571100, China; 4 Hainan Institute for Food Control, Key Laboratory of Tropical Fruits and Vegetables Quality and Safety for State Market Regulation, Haikou 570311, China; 5 Guangdong Provincial Key Laboratory of Food Quality and Safety, College of Food Science, South China Agricultural University, Guangzhou 510641, China

**Keywords:** UPLC-MS/MS, soybean sprout, quinolones, detection, degradation

## Abstract

Recently, there have been increasing safety concerns about the illegal abuse of quinolone in soybean sprouts. This study aimed to establish an ultra-high performance liquid chromatography-tandem mass spectrometry (UPLC-MS/MS) method for the simultaneous detection of 16 quinolones (QNs) in soybean sprouts, and then reveal their degradation characteristics. The samples were extracted with acetonitrile (with 1% formic acid), purified by a C18 adsorbent, and separated by an ACQUITY UPLC BEH C18 (1.7 μm, 2.1 mm × 100 mm) column. The internal standard method was applied for quantitative determination. The results demonstrated that the quantification linear range for 16 QNs was between 2.0 ng/mL and 50.0 ng/mL. The detection limits were between 0.5 μg/kg and 4.0 μg/kg, and the quantification limits were between 2.0 μg/kg and 20.0 μg/kg. This method was used to screen for quinolones in 50 batches of market soybean sprouts; the obtained results showed good agreement with those of the standard method. It was found that QNs possessed longer degradation half-life (T_1/2_) in the storage stage of soybean sprouts, while they degraded to some extent during the germination stage via active enzyme action. In particular, ciprofloxacin was the most stable QNs with a T_1/2_ of 70.71 d during the storage stage of soybean sprouts. This work not only offers an accurate and efficient QNs residual analysis strategy but also provides a reference for the supervision and management of QNs in foods.

## 1. Introduction

Soybeans are good source of protein, fat, carbohydrates, and other nutrients essential for human health, particularly in developing countries [1]. Soybeans are generally soaked in water and then placed in the warm and humid conditions to germinate in a week, without expensive equipment and technologies [2,3]. Their nutrients, especially isoflavone, and selenium biofortification, are notably improved due to germination [4,5]. However, soybean sprouts are particularly prone to microbial contamination due to their high nutrient content and the warm temperatures and humid conditions needed for their production [3,6]. Various studies have demonstrated that the germination step is the main source of microbial contamination, with 3.0–6.0 log CFU/g microbes in sprouts, which are 2 or 3 logs greater than those observed outside sprouts [7,8]. Recently, antibiotics, especially quinolones such as enrofloxacin and norfloxacin, were reported to be illegally used in the germination of soybean sprouts to inhibit microbes and improve yields [9].

Quinolones (QNs) belong to a family of synthetic antibiotics structurally related to nalidixic acid, first used clinically in animals in the early 1960s [10]. QNs are heavily used in animal farming as feed additives for disease prevention and growth promotion and in aquaculture as antifungal drugs for inhibiting fungal growth [11]. Most of the QNs administered to animals are excreted unchanged in feces and urine. The continuous input of QNs into the environment can disrupt the structure of soil microbial communities and induce the selection and emergence of antimicrobial resistance [12,13]. Moreover, QNs and derivatives can be absorbed by plants, particularly vegetables, and accumulate in their edible parts. In the European Union and China, several of these QNs have been regulated, and maximum residue limits (MRLs) have been issued for animal food, i.e., meat, fish, milk, and eggs [14,15]. However, no regulation has been set for QN residues in soybean sprouts to date. Thus, the detection and monitoring of QNs are urgently needed to provide technical support for the quality and safety supervision of soybean sprouts.

**Figure 1:**
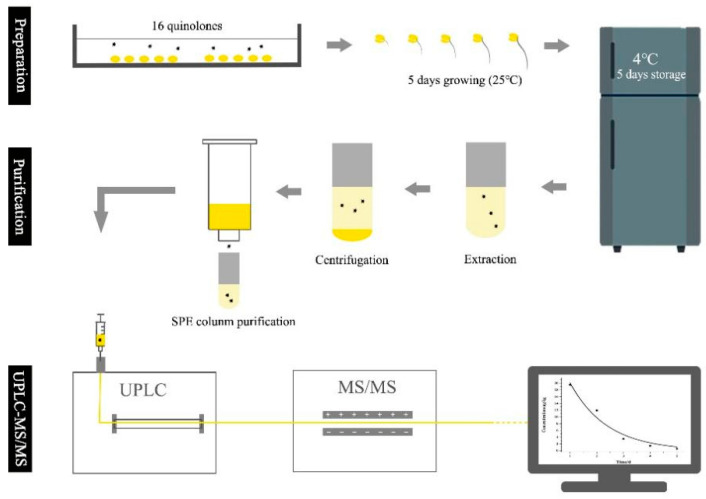
Preparation, purification, UPLC-MS/MS detection of 16 quinolones.

In recent years, novel and reliable methods for the simultaneous determination of multiple QNs have been favored by related researchers. Among these methods, the most widely used analytical method had been liquid chromatography-tandem mass spectrometry (LC-MS/MS); it possesses simple and fast sample pretreatment because of its high sensitivity and selectivity in complex food matrices [16,17]. For example, Liu et al. established LC-MS/MS for detecting enrofloxacin and norfloxacin in bean sprouts [18]. However, the matrix interference of bean sprouts was not completely eliminated due to insufficient purification during sample preparation. Only two QNs can be accurately detected by this method, with recovery ranging from 70% to 91%. In this study, we developed an UPLC-MS/MS method with simple pretreatment for the rapid analysis of 16 QNs (Figure 1). Moreover, soybean sprouts have an active metabolism and microbial growth during the germination stage, and their effect on the degradation of QNs has also been investigated. This study could provide a reference for the rapid screening, batch detection, and enaction of food-safety standards for QNs in soybean sprouts.

## 2. Materials and Methods

### 2.1. Standards and Reagents

Sixteen QNs (100 μg/mL) were purchased from Achemtek Co., Ltd. (Worcester, MA, USA). Ciprofloxacin-D8 (100 μg/mL) was purchased from Dr. Ehrenstorfer (Augsburg, Germany). Enrofloxacin-D5 (100 μg/mL) was purchased from Achemtek Co., Ltd. (Worcester, MA, USA). Norfloxacin-D5 (100 μg/mL) was purchased from Beijing Manhage Bio-Tech Co., Ltd. (Beijing, China).

### 2.2. Sample Preparation

One kilogram of soybeans was soaked in 4 L of water with 75 ng/g QNs for 12 h (CK group were soaked in water without QNs) and covered with a towel, and 200 mL of water was sprayed every 12 h to maintain the towel’s moistness. The soybeans were germinated at 25 °C and 70% humidity for five days without light. The harvested soybean sprouts were then stored in a refrigerator at 4 °C for five days.

### 2.3. Sample Purification

Two-hundred and fifty grams of soybean sprout samples were mixed using a blender for two minutes, and 5.0 g of homogenized soybean sprout samples were placed in a 50 mL centrifuge tube. Then, a 40 μL mixed internal standard solution (10.0 μg/mL), 2 g NaCl, and 20 mL acetonitrile (with 1% formic acid) were added to the samples. After being vortexed for 10 min and sonicated in a water bath for 10 min, the tube was centrifuged at 10,000 r/min for 5 min. Five mL supernatant was purified by Agilent Bond Elut C18 (500 mg, 6 mL), Waters Oasis PRime HLB (200 mg, 6 mL), and Agilent Bond Elut-PSA (500 mg, 6 mL), respectively. There was no evaporation and redissolution after purification using the SPE column. Most of the substances that may interfere with the results were retained in the SPE column, while the retention of QNs and acetonitrile in SPE was nearly negligible. Finally, UPLC-MS/MS analysis was performed after being filtered by SCAA-104 (0.22 μm).

### 2.4. Preparation of Standard Solution

Five grams of soybean sprouts from the CK group were pretreated by the method described above. The filtered supernatant was obtained to prepare a series of standard solutions from 2.0 to 50 ng/mL QNs.

### 2.5. UPLC-MS/MS Analysis

UPLC: Analyses were performed using an UPLC (Ultimate 3000, Thermo, WA, USA) equipped with four columns: Waters ACQUITY UPLC BEH C18 (1.7 μm, 2.1 mm × 100 mm), CORTECS T3 (2.7 μm, 2.1 mm × 100 mm), EclipsePlus RRHD C18 (1.8 μm, 2.1 mm × 100 mm), and Kinetex C8 100A (1.7 μm, 2.1 mm × 100 mm). The column temperature was 35 °C, the injection volume was 5.0 μL, and the flow rate was 0.3 mL/min. Mobile phase A was 0.1% formic acid prepared in water, and mobile phase B was acetonitrile. The mobile phase gradient was programmed as follows: 0~2 min, 5%B; 2~3 min, 5~95%B; 3~7 min, 95%B; and 7.01~10 min, 5%B. The effluent was connected to an ESI-triple quadrupole-linear ion trap (QTRAP)-MS.

MS: ion source: ESI source, positive ion mode; scan mode: selected reaction monitoring (SRM) mode; electrospray voltage: 5500 V; ion source temperature: 550 °C; ion source gas I (GSI), gas II (GSII), curtain gas (CUR) were set at 50, 50, and 25 psi, respectively. The MS parameters of the 16 quinolones are shown in Table S1.

The UPLC-MS/MS standard method of BJS 201909 (the determination of quinolones in soybean products, hotpots, spicy hotpots, and other foods, CN) [19] was used to compare the accuracy of the UPLC-MS/MS method established by us. Briefly, the analyses were performed using the Shimadzu Nexera-LCMS-8050 LC-MS/MS platform equipped with a column (C18, 2.1 mm × 50 mm, 1.7 μm). The column temperature was 40 °C, the injection volume was 2.0 μL, and the flow rate was 0.25 mL/min. Mobile phase A was 5 mmol/L ammonium acetate aqueous solution (containing 0.1% formic acid), and mobile phase B was acetonitrile. The mobile phase gradient was programmed as follows: 0~3 min, 10%B; 3~8 min, 10~30%B; 8~10 min, 30%B; 10~10.5 min, 30~95%B; 10.5~13 min, 95%B; 13~13.1 min, 95~10%B; and 13.1~15 min, 10%B. ESI source: positive ion mode; electrospray voltage: 4000 V; and ion source temperature: 350 °C. Scan mode: multiple reaction monitoring (MRM) mode.

### 2.6. Recovery of Quinolone

Recovery was calculated as: the concentration of QN detected × 20 mL/0.2 μg × 100%.

### 2.7. Statistical Analysis

Adobe Illustrator software (CS4, Adobe Systems Inc., San Jose, CA, USA) and OriginPro software (2019b, OriginLab Inc., Northampton, MA, USA) were used for image processing. SPSS software (version 22.0, SPSS Inc., Armonk, NY, USA) was used to analyze the least significant difference (LSD) at the 5% level.

## 3. Results and Discussion

### 3.1. Optimization of Chromatographic Column and Mobile Phase

Four chromatographic columns and six different mobile phases, including 0.2% formic acid aqueous/methanol, water/methanol, water/acetonitrile, 0.2% formic acid aqueous/acetonitrile, 5 mmol/L ammonium acetate aqueous/acetonitrile, and 5 mmol/L ammonium acetate aqueous/methanol, were used for the UPLC separation of the 16 QNs. Figure S1 shows the best separation for the 16 QNs by Waters of the BEH C18 column with 0.2% formic acid aqueous/acetonitrile as the mobile phase. When the mobile phase solvent contained 0.2% formic acid, the 16 QNs were better separated by UPLC with symmetrical peak shape, high signal response, and good stability of MS/MS. This might be related to the more stable ionization of QNs under acidic conditions.

### 3.2. Optimization of Extraction Solvent

Since most QNs are amphoteric, the pH of the extraction solvent can significantly affect the analyte recovery [19]. The extraction efficacy of acetonitrile solutions containing different concentrations of formic acid (0~5%) was evaluated. As shown in Figure S2, the recoveries of pefloxacin, flurofloxacin, sparfloxacin, and orbifloxacin extracted with pure acetonitrile were 210.8%, 198.9%, 362.4%, and 277.3%, respectively, indicating that the matrix effect was high. The recoveries of most QNs were improved by adding formic acid, but low recoveries were still observed for some QNs. Although acidic solvents enhanced the extraction of QNs from food samples [20], strong acidic conditions can also promote the extraction of other components, which can interfere the accurate quantification. In addition, the formation of ion pairs between the protonated structures of the QNs and acid anions can also account for the unacceptable recoveries (higher than 120% or less than 70%) [21]. Among the six extraction solvents, acetonitrile with 1% formic acid could eliminate the matrix effect to acceptable levels with recoveries of 16 QNs ranging from 82.9% to 117.8%, exhibiting the optimal extraction efficacy. Therefore, acetonitrile with 1% formic acid was chosen as the optimal extraction solvent for further experiments.

### 3.3. Optimization of SPE Purification Column

Subjecting the extract solution to solid-phase extraction (SPE) is a commonly used pretreatment procedure for QN detection. The decisive factor for the purification efficiency of SPE is the sorbent, and various types of sorbent materials have been investigated for the extraction and concentration of QNs from samples [19]. Three SPE purification columns with different sorbent materials, namely, C18, PRime HLB, and PSA, were compared. As shown in Figure S3, only six QNs, including enrofloxacin, sarafloxacin, and difluoxacin were recovered from 92.5% to 214.3% when using a PSA column. When using a PRime HLB column, the recovery of sparfloxacin was as high as 152.3%. However, satisfactory recoveries for all 16 QNs (76.5~108.7%) were attained when using C18 column for purification. Therefore, the C18 column was selected for the pretreatment of soybean sprouts, due to its excellent matrix effect removal efficacy.

### 3.4. Sensitivity and Quantification Accuracy of the 16 Quinolones by UPLC-MS/MS

Under optimal conditions, UPLC-MS/MS was performed to detect 16 QNs. As shown in Table 1, all 16 QNs can be quantified in the linear range from 2.0 ng/mL to 50 ng/mL. The correlation coefficient R^2^ of 16 calibration curves was higher than 0.99849. The limits of detection (LOD) and the limits of quantification (LOQ) of the method were determined by means of instrumental signal-to-noise ratio rations of 3 and 10, respectively. The LOD was between 0.5 μg/kg and 4.0 μg/kg, LOQ was between 8.0 μg/kg and 20.0 μg/kg. The LOD was lower than the standard method (BJS 201909), according to which the LOD for QNs is 5 μg/kg. The relative standard deviations (RSDs) of the method ranged from 1.6% to 7.9% for the six replicate determinations of the 16 QNs at a concentration of 10.0 ng/mL, indicating the proposed method had good precision. Subsequently, a recovery test was performed by spiking 16 QNs at 8, 40, and 80 μg/kg concentration levels in the CK group. The average recovery was 75.7~119.4% (*n* = 6). These results indicated the good sensitivity and accuracy of the UPLC-MS/MS.

### 3.5. Screening of Quinolones in Soybean Sprouts from Market

In order to verify the reliability of the UPLC-MS/MS method established above, we purchased 50 soybean sprout samples from the market in Haikou and analyzed the residues of 16 QNs. The results were compared and verified by the standard method (BJS 201909). As shown in Table 2, three samples tested positive, including 30.5 μg/kg of enrofloxacin, 120.0 μg/kg of ciprofloxacin, and 33.9 μg/kg of norfloxacin, which are consistent with the results of standard method. According to the national food safety standard of China, enrofloxacin, ciprofloxacin, and other QNs must not be detected in foods. The above results revealed the potential risk of QN residues in soybean sprouts, showing that the monitoring of QN residues in soybean sprouts needs to be strengthened.

### 3.6. Degradation Characterization of 16 Quinolones in Production Stage of Soybean Sprouts

To investigate the degradation characteristics of QNs, we simulated the illegal production of sprouts by adding 16 QNs to soybean to produce sprouts and monitored the variation in QN residue levels. The soybeans were germinated at room temperature for five days, followed by five days of storage at 4 °C. The concentrations of the 16 QNs during production and storage were analyzed by UPLC-MS/MS. The first-order kinetic models described in [22] were applied to characterize the degradation kinetics of 16 QNs; the results are shown in Table 3. The R^2^ values of degradation equations for the 16 QNs ranged from 0.7618 to 0.9816, and the degradation half-life (T_1/2_) of the QNs ranged from 0.96 d to 16.38 d. Interestingly, the half-lives of orbitroxacin, fleroxacin, and lomefroxacin were longer than the others, which were 16.38 d, 13.03 d, and 10.21 d, respectively. Unlike the other 13 QNs, these three QNs have a fluorine atom in the C8 position, which increases their absorption and T_1/2_ [23].The degradation of nalidixic acid, oxalic acid, and flumequine was faster, with degradation half-lives of 0.96 d, 1.5 d and 1.54 d, respectively, which can be ascribed to the absence of a piperazine ring in position C7 of the quinolone nucleus for these three QNs, thus resulting in relatively lower stability of these molecules [24].

### 3.7. Degradation Characterization of 16 Quinolones in Soybean Sprouts during Storage

Since the stability of quinolones in soybean sprouts stored at 4 °C has not been reported [25], we first investigated the degradation kinetics of QNs during the storage stage of soybean sprouts. Compared with the sprout growth stage, the T_1/2_ of 15 QNs increased during the storage stage, the exception being fleroxacin. Among them, the half-lives of ciprofloxacin, danofloxacin and sparfloxacin were longer than others, at 70.71 d, 52.5 d, and 31.5 d, respectively. However, sparfloxacin degraded rapidly during the soybean sprouts’ growth stage, and its T_1/2_ was only 2.9 d. In contrast, sparfloxacin was more stable when stored at 4 °C, and its T_1/2_ extended to 31.5 d. This phenomenon can be attributed to the vigorous enzyme action which boosts the demand for nitrogen sources during the growth stage of soybean sprouts. Dikshit et al. reported that the availability of free amino acids and amino nitrogen content increased four- to eight-folds during germination, respectively [26]. Thus, the -NH_2_ in the C6 position was prone to be exhausted as a nitrogen source, expediting the degradation of sparfloxacin. Notably, nalidixic acid was completely degraded on the fourth day of the storage stage. Since nalidixic acid is a type of antibiotic whose use is prohibited in soybean sprouts, it should be sampled and detected as soon as possible to ensure accuracy.

A substance’s degradation is not only determined by the compound itself but also by test conditions such as temperature and light intensity. In this study, the T_1/2_ of ofloxacin in the production and storage stage of soybean sprouts was 7.59 d and 11 d, respectively, significantly different to the results reported by Alexy et al. [27]. Alexy et al. monitored the degradation of ofloxacin in a closed bottle kept in the dark at room temperature (20 ± 1 °C) and found ofloxacin degraded by less than 60% after 28 days. Thus, ofloxacin was deemed to be not readily biodegradable [28]. Additionally, it should be noted that the matrix also plays an essential role in determining the stability of antibiotics. The same antibiotic dissolved in different matrices, such as animal plasma, tissues, foodstuffs, or sample extracts, may exhibit different levels of stability [29,30]. In this study, the degradation characterization of the 16 QNs in soybean sprouts was revealed, which could provide a reference for the degradation of QNs in other food matrixes.

## 4. Conclusions

In this study, we established an UPLC-MS/MS with good sensitivity, accuracy, and reliability for the simultaneous detection of 16 QNs in soybean sprouts. The soybean sprout samples were extracted with acetonitrile (with 1% formic acid) and purified using an C18 SPE column. Separation was performed on an ACQUITY UPLC BEH C18 (1.7 μm, 2.1 mm × 100 mm) column. The internal standard method was used for quantitative calculation. The UPLC-MS/MS was used to screen QNs in 50 batches of soybean sprouts from a market and revealed the potential risk of QN residues in soybean sprouts. Moreover, the degradation characteristics of 16 QNs during the production and storage stage of soybean sprouts were investigated for the first time using this method. It was found that QNs commonly possessed longer degradation half-lives in the storage stage of soybean sprouts, while they degraded to some extent during the germination stage via active enzyme action. Among the 16 QNs, ciprofloxacin possessed the highest stability, with a T_1/2_ of 70.71 d during the storage stage. Nalidixic acid, a prohibited drug, completely decomposed on the fourth day of storage stage. Given this, we appeal to related agencies and departments to strengthen the supervision on the illegal usage and residual risk of QNs in soybean sprouts.

## Figures and Tables

**Table 1:** Linear equation, limits of detection and quantification, precision of 16 quinolones.

Compound	Linear Equation	R^2^	Limit of Detection/(μg/kg)	Limits of Quantification/(μg/kg)	RSD/% (*n* = 6)
Enrofloxacin	y = 1.08828x + 0.01962	0.99958	4.0	8.0	4.3
Ciprofloxacin	y = 1.54110x + 0.09348	0.99849	2.0	8.0	2.2
Ofloxacin	y = 0.96849x + 0.04587	0.99901	4.0	8.0	2.3
Norfloxacin	y = 1.39138x − 0.03124	0.99947	3.0	8.0	7.1
Pefloxacin	y = 0.67444x + 0.03205	0.99875	1.0	8.0	5.2
Lomefloxacin	y = 4.53358x − 0.05440	0.99956	4.0	20.0	6.0
Dalfloxacin	y = 4.07682x + 0.00796	0.99870	3.0	4.0	4.3
Sarafloxacin	y = 1.00208x − 0.00294	0.99938	1.0	20.0	6.4
Flurofloxacin	y = 0.93473x + 0.10372	0.99912	2.0	20.0	7.9
Difluoxacin	y = 1.24093x + 0.04245	0.99926	3.0	20.0	6.5
Sparfloxacin	y = 1.58249x − 0.04456	0.99922	1.0	8.0	5.6
Enoxacin	y = 1.76071x − 0.00857	0.99943	2.0	20.0	5.9
Nalidixic acid	y = 71.34290x + 1.22451	0.99905	0.5	2.0	2.3
Oxalic acid	y = 70.85536x − 1.20356	0.99951	1.0	2.0	2.8
Flumequine	y = 74.88015x − 0.62798	0.99923	1.0	2.0	1.6
Orbifloxacin	y = 1.30092x + 0.13993	0.99910	1.0	8.0	7.4

**Table 2:** Comparison and verification of the two analysis methods.

Compound	UPLC-MS/MS Method Established by Us (ug/kg)	UPLC-MS/MS Method of BJS 201909 (ug/kg)	Number of Positive Samples
Enrofloxacin	30.5 ± 0.2	31.7 ± 0.3	1
Ciprofloxacin	120.0 ± 0.8	124.1 ± 0.8	1
Norfloxacin	33.9 ± 0.1	35.1 ± 0.2	1

**Table 3:** Degradation characterization of 16 QNs in production and storage stage of soybean sprout.

Compound	Production Stage					Degradation Equation	R^2^	T_1/2_	Storage Stage					Degradation Equation	R^2^	T_1/2_
	1 d	2 d	3 d	4 d	5 d				6 d	7 d	8 d	9 d	10 d			
Orbifloxacin	21.6	20.5	20.2	19.6	17.8	C = 22.5950 × e^−0.0423t^	0.9143	16.38	16.7	16.6	16.4	15.6	15.3	C = 17.2841 × e^−0.0234t^	0.9035	29.62
Fleroxacin	20.9	17.8	17.9	17.9	16.1	C = 21.193 × e^−0.0532t^	0.7618	13.03	12.8	12.9	12.4	10.9	10.3	C = 14.0585 × e^−0.0578t^	0.8546	11.99
Lomefloxacin	20.9	19.6	17.7	17.1	16.0	C = 22.2827 × e^−0.0679t^	0.9816	10.21	13.0	12.6	12.2	11.5	11.2	C = 13.573 × e^−0.0388t^	0.9848	17.86
Norfloxacin	20.8	19.3	18.3	18.1	13.7	C = 22.9867 × e^−0.083t^	0.8196	8.35	12.8	12.6	11.5	10.7	10.4	C = 13.7478 × e^−0.0577t^	0.9546	12.01
Enrofloxacin	20.4	17.9	17.0	15.9	14.2	C = 21.8759 × e^−0.0849t^	0.9755	8.16	12.2	11.9	11.5	10.9	9.9	C = 13.0324 × e^−0.0489t^	0.9284	14.17
Enoxacin	21.5	20.3	19.2	16.9	14.8	C = 24.0252 × e^−0.089t^	0.956	7.79	13.8	13.4	12.6	11.4	10.8	C = 14.9644 × e^−0.064t^	0.9678	10.83
Ofloxacin	21.7	16.5	16.3	16.2	14.4	C = 22.1889 × e^−0.0913t^	0.7644	7.59	13.4	12.8	12.1	10.7	10.7	C = 14.3661 × e^−0.063t^	0.9443	11.00
Pefloxacin	21.3	20.9	17.8	17.3	13.6	C = 24.4381 × e^−0.102t^	0.9019	6.79	13.4	12.5	10.3	10.0	10.0	C = 14.4115 × e^−0.0853t^	0.8731	8.12
Difloxacin	20.8	18.2	16.8	15.9	13.1	C = 22.9377 × e^−0.1042t^	0.9666	6.65	10.0	9.9	9.4	9.1	9.1	C = 10.3084 × e^−0.0275t^	0.9171	25.20
Sarafloxacin	19.9	17.0	16.0	15.1	11.6	C = 22.2009 × e^−0.1152t^	0.932	6.02	9.5	9.3	8.6	8.4	7.9	C = 10.0362 × e^−0.0468t^	0.9687	14.81
Ciprofloxacin	19.5	19.6	16.1	13.5	12.8	C = 22.9318 × e^−0.1184t^	0.9117	5.85	11.6	11.3	11.3	11.2	11.1	C = 11.6347 × e^−0.0098t^	0.8666	70.71
Danofloxacin	24.5	23.8	19.7	16.4	15.8	C = 28.5983 × e^−0.1236t^	0.9391	5.61	15.6	15.5	15.2	14.9	14.9	C = 15.830 × e^−0.0132t^	0.9357	52.50
Sparfloxacin	21.5	15.5	11.5	9.8	9.6	C = 26.205 × e^−0.2392t^	0.9421	2.90	8.5	8.3	8.2	8.1	7.7	C = 8.7112 × e^−0.022t^	0.9159	31.50
Flumequine	21.0	19.1	7.7	4.8	2.5	C = 35.8092 × e^−0.4513t^	0.8978	1.54	1.9	1.8	1.5	1.4	1.0	C = 2.272 × e^−0.1403t^	0.9223	4.94
Oxalic acid	18.9	16.4	8.5	4.2	1.1	C = 32.187 × e^−0.4521t^	0.9143	1.50	1.1	1.1	1.0	1.3	1.4	C = 0.9477 × e^0.0713t^	0.6231	----
Nalidixic acid	19.5	11.9	3.5	1.4	0.6	C = 41.3012 × e^−0.7202t^	0.9714	0.96	0.2	0.2	0.2	0.0	0.0	C = 0.3474 × e^−0.3877t^	0.6007	1.79

Note: according to the degradation equation and corresponding parameters simulated by the first-order kinetic model C = C_0_ × e^-kt^ (C is the concentration on day t, μg/kg; C_0_ is the initial concentration, μg/kg; t is the time, d; k is the degradation rate constant). The half-lives of quinolones are expressed as T_1/2_ (At this time, C = 1/2 C_0_, T_1/2_ = ln2·k^−1^ = 0.693·k^−1^). ---- means no T_1/2_.

## Data Availability

All data presented in the present research are available on request from the corresponding author.

## Supplementary Materials

The following supporting information can be downloaded at: https://www.mdpi.com/article/10.3390/foods11162500/s1, Table S1: MS parameters of 16 Quinolones; Figure S1: The 16 quinolones of MRM chromatogram; Figure S2: The effect of extraction solvents on the recoveries of 16 quinolones; Figure S3: The effect of purification of column on the recoveries of 16 quinolones.
