# Supplementary material for: Detection and Degradation Characterization of 16 Quinolones in Soybean Sprouts by Ultra-High Performance Liquid Chromatography-Tandem Mass Spectrometry
Source: Foods. 2022 Aug 18;11(16):2500. doi: 10.3390/foods11162500 (PMC9407237; doi:10.3390/foods11162500)
Supplement: Supplementary file 1 [file foods-11-02500-s001.zip › foods-1824602-supplementary.pdf]

**Table S1.** MS parameters of 16 Quinolones

| Compound | Time/ min | Parent ion mass to charge ratio/(m/z) | Quantitative mass to charge ratio/(m/z) | Qualitative mass to charge ratio/(m/z) | Collision energy/V | Debunching voltage/V | Internal standard |
|---|---|---|---|---|---|---|---|
| Enrofloxacin | 3.39 | 360.3 | 316.2 | 245.1 | 24/36 | 100 | Enrofloxacin-D5 |
| Ciprofloxacin | 3.28 | 332.2 | 314.1 | 231.0 | 30/30 | 100 | Ciprofloxacin-D8 |
| Ofloxacin | 3.25 | 362.2 | 318.1 | 261.1 | 26/38 | 100 | Norfloxacin-D5 |
| Norfloxacin | 3.23 | 320.2 | 302.2 | 233.1 | 35/33 | 100 | Norfloxacin-D5 |
| Pefloxacin | 3.27 | 334.3 | 316.1 | 290.0 | 26/24 | 108 | Norfloxacin-D5 |
| Lomefloxacin | 3.34 | 352.2 | 265.1 | 334.1 | 32/30 | 100 | Ciprofloxacin-D8 |
| Dalfloxacin | 3.32 | 358.2 | 340.1 | 255.0 | 31/53 | 110 | Ciprofloxacin-D8 |
| Sarafloxacin | 3.55 | 386.2 | 299.0 | 368.2 | 28/31 | 115 | Enrofloxacin-D5 |
| Flurofloxacin | 3.27 | 370.2 | 326.1 | 269.0 | 24/38 | 100 | Norfloxacin-D5 |
| Difluoxacin | 3.57 | 400.2 | 356.2 | 382.1 | 39/31 | 110 | Ciprofloxacin-D8 |
| Sparfloxacin | 3.55 | 393.2 | 349.2 | 292.0 | 26/35 | 120 | Enrofloxacin-D5 |
| Enoxacin | 3.23 | 320.2 | 302.1 | 233.1 | 24/34 | 100 | Norfloxacin-D5 |
| Nalidixic acid | 4.39 | 233.1 | 215.0 | 187.1 | 18/34 | 60 | Enrofloxacin-D5 |
| Oxalic acid | 3.99 | 262.1 | 244.1 | 160.1 | 23/47 | 80 | Enrofloxacin-D5 |
| Flumequine | 4.44 | 262.2 | 244.0 | 202.1 | 23/25 | 80 | Enrofloxacin-D5 |
| Orbifloxacin | 3.43 | 396.2 | 295.1 | 352.0 | 34/24 | 103 | Ciprofloxacin-D8 |
| Ciprofloxacin-D8 | 3.27 | 340.3 | 322.1 | --- | 28 | 50 | --- |
| Enrofloxacin-D5 | 3.39 | 365.1 | 321.1 | --- | 26 | 50 | --- |
| Norfloxacin-D5 | 3.23 | 325.3 | 307.2 | --- | 42 | 110 | --- |

Note: --- means no internal standard.

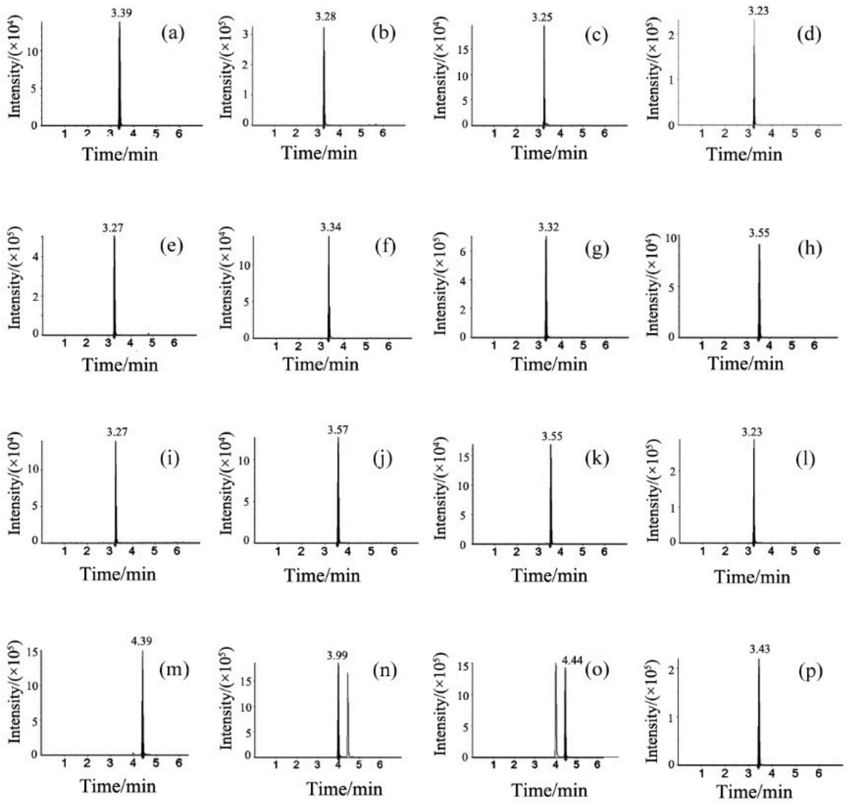


**Figure S1.** The 16 quinolones of MRM chromatogram
(a)-Enrofloxacin; (b)-Ciprofloxacin; (c)-Ofloxacin; (d)-Norfloxacin; (e)-Pefloxacin; (f)-Lomefloxacin; (g)-Dalfloxacin; (h)-Sarafloxacin; (i)-Flurofloxacin; (j)-Difluoxacin; (k)-Sparfloxacin; (l)-Enoxacin; (m)-Nalidixic acid; (n)-Oxalic acid; (o)-Flumequine; (p)-Orbifloxacin

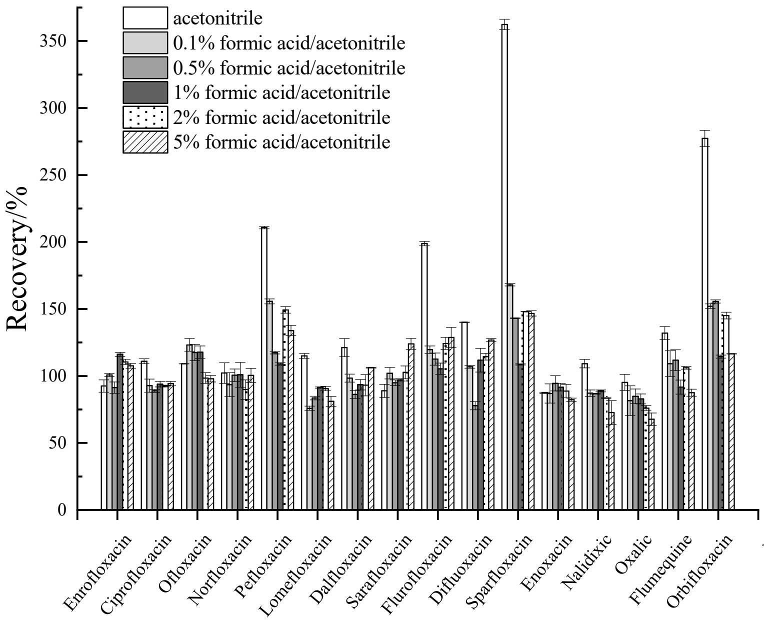


**Figure S2.** The effect of extraction solvents on the recoveries of 16 quinolones

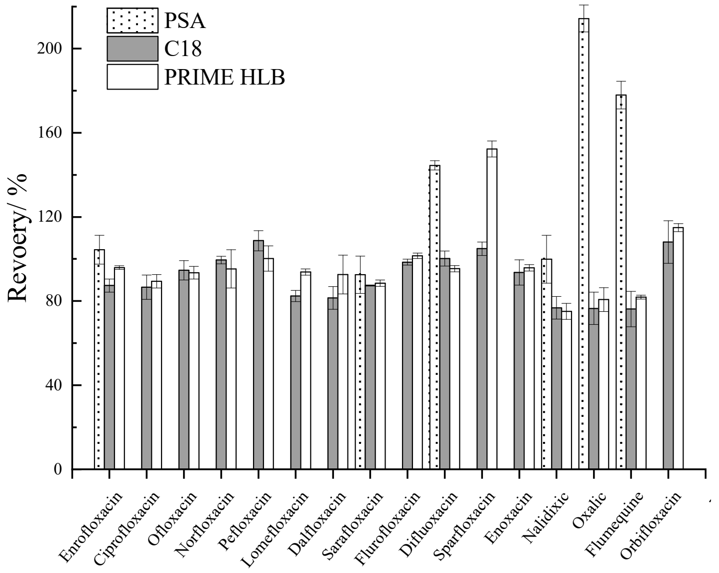


**Figure S3.** The effect of purification of column on the recoveries of 16 quinolones
